# Characteristics of Immune Checkpoint Inhibitor-Associated Gastritis: Report from a Major Tertiary Care Center

**DOI:** 10.1093/oncolo/oyad031

**Published:** 2023-03-11

**Authors:** Natalie Farha, Muhammad Salman Faisal, Daniela S Allende, Joseph Sleiman, Ravi Shah, Nicole Farha, Pauline Funchain, Jessica R Philpott

**Affiliations:** Department of Internal Medicine, Cleveland Clinic, Cleveland, OH, USA; Department of Internal Medicine, Cleveland Clinic, Cleveland, OH, USA; Pathology Department, Cleveland Clinic, Cleveland, OH, USA; Department of Internal Medicine, Cleveland Clinic, Cleveland, OH, USA; Department of Internal Medicine, Cleveland Clinic, Cleveland, OH, USA; Department of Internal Medicine, Cleveland Clinic, Cleveland, OH, USA; Department of Hematology and Oncology, Taussig Cancer Institute, Cleveland Clinic, Cleveland, OH, USA; Department of Gastroenterology, Hepatology and Nutrition, Cleveland Clinic, Cleveland, OH, USA

## Abstract

**Background:**

Immune checkpoint inhibitors (ICIs) have increased our ability to treat an ever-expanding number of cancers. We describe a case series of 25 patients who were diagnosed with gastritis following ICI therapy.

**Materials and Methods:**

This was a retrospective study involving 1712 patients treated for malignancy with immunotherapy at Cleveland Clinic from January 2011 to June 2019 (IRB 18-1225). We searched electronic medical records using ICD-10 codes for gastritis diagnosis confirmed on endoscopy and histology within 3 months of ICI therapy. Patients with upper gastrointestinal tract malignancy or documented *Helicobacter pylori*-associated gastritis were excluded.

**Results:**

Twenty-five patients were found to meet the criteria for diagnosis of gastritis. Of these 25 patients, most common malignancies were non–small cell lung cancer (52%) and melanoma (24%). Median number of infusions preceding symptoms was 4 (1-30) and time to symptom onset 2 (0.5-12) weeks after last infusion. Symptoms experienced were nausea (80%), vomiting (52%), abdominal pain (72%), and melena (44%). Common endoscopic findings were erythema (88%), edema (52%), and friability (48%). The most common diagnosis of pathology was chronic active gastritis in 24% of patients. Ninety-six percent received acid suppression treatment and 36% of patients also received steroids with an initial median dose of prednisone 75 (20-80) mg. Within 2 months, 64% had documented complete resolution of symptoms and 52% were able to resume immunotherapy.

**Conclusion:**

Patients presenting with nausea, vomiting, abdominal pain, or melena following immunotherapy should be assessed for gastritis and if other causes are excluded, may require treatment as consideration for complication of immunotherapy.

## Teaser

This article describes a case series of 25 patients who were diagnosed with gastritis following immune checkpoint inhibitor therapy.

**Implications for Practice**

This report contributes to the identification and management of this pathology by providing data on a large collection of cases describing gastritis in the setting of immune checkpoint inhibitor (ICI) therapy. This report provides a complete clinicopathological picture rather than focusing on one aspect alone for clinicians to recognize the disease pattern of ICI gastritis. The results of this study are clinically relevant as they will allow prescribers to identify and treat patients with ICI-induced gastritis early to minimize interruption of immune checkpoint inhibitor therapy.

## Introduction

Over the last decade, the emergence of immune check-point inhibitor (ICI) therapy has revolutionized the treatment of a growing number of malignancies.^1^ Enhancement of immune response against tumor antigens underlies the mechanism of action of these drugs. This is achieved by blockade of co-inhibitory receptors on T-cells mainly cytotoxic T-lymphocyte antigen 4 (CTLA-4), programmed cell death protein 1 (PD-1), and programmed-death ligand 1 (PDL-1).^2^ Downregulation of T-cell response by these co-inhibitory receptors is desirable in certain situations such as recognition of self-antigens. However, tumors often express checkpoint proteins that inactivate T cells using this mechanism allowing tumor cells to evade the immune system.^3^ Blocking these receptors prevents downregulation of T cells and antitumor action.

Ipilimumab, a CTLA-4 inhibitor was the first drug of this class to be approved for the treatment of malignant melanoma in 2011.^4^ It was followed by other drugs, PD1/PD-L1 inhibitors such as nivolumab, pembrolizumab, and atezolizumab which have gained significant importance in treatment of many cancers due to better tolerability and superior efficacy.^5^ Aside from melanoma, they are used alone or in combination for treatment of a growing list of cancers such as lung, ovarian, renal, and prostate cancers.^6-8^ Various clinical trials are underway to evaluate the use of ICI therapy for other malignancies.

The upregulation of immune system by these medications can lead to a loss of self-tolerance. This can lead to various toxicities commonly referred to as immune-related adverse events (irAEs). The most common organs affected are gastrointestinal tract, liver, musculoskeletal system, endocrine organs, skin, and lungs. These gastrointestinal toxicities are one of the most common, and colitis in this regard has been extensively described for both CTLA-4 and PD-1/PD-L1 inhibitors. However, to date, there are numerous case reports/series but only a few observational studies that describe upper gastrointestinal toxicity related to the use of these medications, particularly gastritis, outlined in Table 1.^9-12^

**Table 1.** Review of immune checkpoint inhibitor-induced gastritis literature.

Author Year Country	Study type	Population	Symptoms	Histologic findings
Collins^13^ 2017 France	Retrospective cohort	20 patients with confirmed GI irAE, 4 patients had gastritis and EGD	4 (100%) had nausea/vomiting 3 (75%) had abdominal pain 2 (59%) had diarrhea	1/4 (25%) had a *H. pylori*-related gastritis 1/4 (25%) had a non-specific gastritis 2/4 (50%) had increased intraepithelial lymphocytes
Tang^12^ 2019 US	Observational	60 patients with ICI-induced gastritis who had an EGD	47 (78.3%) had nausea/vomiting 17 (28%) had epigastric pain 11 (18%) had upper GI tract bleeding with anemia	41 (68%) had abnormal EGD findings. –34 (56.7%) had non-ulcerative inflammation –19 (31.7%) had normal appearance –7 (12%) had mucosal ulceration
Johncilla^9^ 2020 US	Observational	12 patients with irAE gastritis	7 (58.3%) had nausea/vomiting 7 (58.3%) had diarrhea, all of whom also had concomitant inflammation of duodenum or colon	8 (66.7%) had chronic active gastritis 4 (33.3%) had focal enhancing gastritis 7 (58.3) had increased intra-epithelial lymphocytes
Zhang^10^ 2020 US	Observational	39 patients with GI irAE and upper GI biopsies	16 (41%) had nausea/vomiting 32/39 had diarrhea	15 (38%) had inflammation in the gastric pit/isthmus/neck region, termed peri-gland disease 12 (31%) had non-necrotizing granulomas 5 (12.8%) had active gastritis with neutrophilic abscesses 5 (12.8%) had prominent reactive gastropathy
Present Study 2023 US	Retrospective	25 patients with ICI-induced gastritis who had an EGD	20 (80%) had nausea/vomiting 18 (72%) had had abdominal pain 13 (52%) had diarrhea	22 (88%) had erythema 13 (52%) had edema 12 (48%) had friability Slides reviewed by a GI pathology specialist (*n* = 14) 5 (35.7%) had chronic gastritis 4 (28.6%) reactive gastropathy

Abbreviations: GI, gastrointestinal; irAE, immune-related adverse events; ICI, immune checkpoint inhibitors; EGD, esophagogastroduodenoscopy.

To attempt to add to the understanding of this complication of immune therapy, we present a detailed case series with information about the clinical presentation, endoscopic features, histology, treatment, and outcome of gastritis in the setting of ICI use.

## Materials and Methods

This was a retrospective study involving 1712 patients treated for malignancy with ICI therapy at Cleveland Clinic from January 2011 to June 2019. Gastritis cases were identified using electronic medical records to search for ICD-10 code for gastritis within 3 months of last immunotherapy transfusion. Eighty-one patients were identified and we then carried out a manual chart review to confirm gastritis cases based on the following clinical definition; the presence of symptoms such as nausea, vomiting, epigastric pain, weight loss, or melena along with objective evidence of gastric inflammation on subsequent endoscopy. Patients with upper gastrointestinal tract malignancy or documented *Helicobacter pylori*-associated gastritis were excluded to identify 25 patients.

Data were collected from medical chart review for patient demographics, type of malignancy, class of drug and duration of ICI therapy preceding gastritis, time to symptoms from last infusion, presenting signs, and symptoms. Data regarding co-existence of other complications of ICI therapy were collected. We also collected data regarding treatment strategies used by providers, including use of acid suppression treatment and corticosteroids, resolution of symptoms, and continuation of ICI therapy. We also evaluated for other potential causes of gastritis such as concurrent NSAID use, *H. pylori*, and chemotherapy or radiation within 3 months of presentation.

A gastrointestinal pathologist reviewed the biopsy slides that were available. H&E stained sections from 14 cases were reviewed for the following parameters: predominant pattern of injury which was further classified as (i) chronic gastritis (active or inactive), when an increased lymphoplasmacytic inflammatory infiltrate was present in lamina propria (LP), (ii) focal enhancing gastritis which was defined as foci of neutrophilic infiltrate within the epithelium without diffuse lymphoplasmacytic inflammation of LP, (iii) reactive gastropathy defined as smooth muscle proliferation in the LP, corkscrew shaped foveolar hyperplasia and mucin depletion in surface foveolar epithelium, and (iv) lymphocytic gastritis, and (v) acute gastritis pattern of injury. Disease activity was classified as mild (rare neutrophils within the gland epithelium), moderate (gland lumen contains neutrophilic abscesses), and severe (mucosal erosions/ulceration). We also evaluated for degree of LP inflammation (none, mild, moderate, and severe), the presence of lymphoid aggregates, and apoptosis. A morphologic assessment for viral inclusions was done on all specimens. Immunostains were performed in cases with suspicious morphologic findings.

All data are presented as numbers and percentages. Approval for the study was obtained after review by Cleveland Clinic institutional review board (IRB 18-1225).

## Results

### Patient Population

Mean age at the time of diagnosis of ICI-associated gastritis was 64.80 ± 11.1 years. We had a higher number of males 15 (60%) compared to females 10 (40%). Our patients were predominantly Caucasian 20 (80%). Non–small cell lung cancer (52%) and melanoma (24%) were the most common indications for immunotherapy. Colon (*n* = 2, 8%), urothelial (*n* = 2, 8%), mesothelioma (*n* = 1, 4%), and non-Hodgkin’s lymphoma (*n* = 1, 4%) were the other malignancies seen in this group. Table 2 shows the demographic characteristics and details the various cancers and immunotherapies used in our population.

**Table 2.** Summary of demographics and details of cancer and exposure.

Baseline characteristics	*N* = 25
Age, mean ± SD	64.80 ± 11.1
Gender-male (%)	15 (60.0)
Race, no. of patients (%)	
White	20 (80.0)
Black	3 (12.0)
Others	2 (8.0)
Type of cancer, no. of patients (%)	
Melanoma	6 (24.0)
Lung	13 (52.0)
Colon	2 (8.0)
Urothelial	2 (8.0)
Mesothelioma	1 (4.0)
Non-Hodgkins lymphoma	1 (4.0)
Immunotherapy regimens, no. of patients (%)	
Pembrolizumab	10 (40.0)
Ipilimumab + nivolumab	4 (16.0)
Nivolumab	9 (36.0)
Atezolizumab	1 (4.0)
Durvalumab	1 (4.0)
Mean duration of treatment before symptoms	
Number of infusions, mean ± SD	7.73 ± 7.2
Median (min-max)	4 (1-30)
Time to symptoms after last dose	
Weeks, mean ± SD	2.23 ± 2.3
Median (min-max)	2.0 (0.5-12)
Prior history of documented gastritis	3 (11.5)
Other irAEs	12 (46.2)
Colitis	8 (32.0)
Arthritis	2 (8.0)
Nephritis	1 (4.0)
Hepatitis	2 (8.0)
Pancreatitis	1 (4.0)
Possible gastritis etiologies other than immunotherapy, no. of patients (%)	11 (44.0)
Chemotherapy within 3 months	7 (28.0)
Radiation within 3 months	6 (24.0)
NSAID within a week	1 (4.0)

Abbreviations: irAE, immune-related adverse events; NSAID, non-steroidal anti-inflammatory drugs.

Twenty-five (1.4%) patients were identified with ICI-related gastritis based on the above definition from 1762 individuals. Four (16%) patients received combination of ipilimumab and nivolumab and 1 (4%) patient each received durvalumab and atezolizumab each. Duration of immunotherapy before symptom onset was variable with a median number of 4 infusions preceding symptoms ranging between 1 and 30 infusions. Time to symptom was onset following the last immunotherapy transfusion was a median of 2.0 (0.5-12) weeks.

Three (12%) patients had a history of gastritis in their charts prior to the onset of immunotherapy. In 2 (8%) patients, it was attributed to non-steroidal anti-inflammatory drugs (NSAID) use and in 1 had a previous history of *H. Pylori* infection that was treated. Eleven (44%) patients had other potential causes of gastritis. Seven (28%) had received chemotherapy with various agents within 6 months of gastritis presentation. Six (24%) had received radiation with 3 (12%) patients receiving combination of chemotherapy and radiation. One (4%) had documented long-term NSAID use prior to gastritis presentation.

### Clinical Presentation

The most commonly reported symptoms were nausea and abdominal pain (20 (80%)) and (18 (72%)) respectively. Other symptoms experienced were vomiting (13 (52%)), diarrhea (11 (40%)), anorexia (5 (20%)), and bloating (3 (12%)). Of note, melena was reported in 11 (44%) patients. On review of labs, most patients were anemic at the time of gastritis diagnosis with mean hemoglobin of 9.83 ± 2.3 g/dL. They also had low albumin with a mean of 3.38 ± 0.7 g/dL. Mean CRP was elevated at 5.94 ± 4.6. Table 3 summarizes the clinical presentation of our group.

**Table 3.** Summary of the clinical presentation, endoscopic findings, and treatments.

Symptoms on presentation, no. of patients (%)	*N* = 25
Nausea	20 (80.0)
Vomiting	13 (52.0)
Abdominal pain	18 (72.0)
Bloating	3 (12.0)
Anorexia	5 (20.0)
Melena	11 (44.0)
Diarrhea	11 (44.0)
Pertinent labs, mean ± SD	
Hemoglobin, mg/dL	9.83 ± 2.3
Albumin, g/dL	3.38 ± 0.7
C-reactive protein, mg/dL (*n* = 8)	5.94 ± 4.6
Vitamin B12 (*n* = 8)	604.4 ± 353.6
Endoscopy performed, no. of patients (%)	25 (96.2)
Erythematous mucosa	22 (88.0)
Erosions	9 (36.0)
Ulcerations	5 (20.0)
Edema	13 (52.0)
Granularity	5 (20.0)
Friability	12 (48.0)
Atrophy	1 (33.3)
Bleeding signs	8 (32.0)
Focal inflammation	9 (36.0)
Pathology available, no. of patients (%)	16 (64.0)
Acute gastritis	3 (12.0)
Chronic active gastritis	6 (24.0)
Chronic inactive gastritis	4 (16.0)
Reactive gastropathy	3 (12.0)
Treatments, no. of patients (%)	
PPI	24 (96.0)
Sucralfate	10 (40.0)
Anti-nausea	12 (48.0)
Prednisone	9 (36.0)
Prednisone initial m, mg—median (Min-max)	75 (20-80)
Immunotherapy continued after gastritis episode, no. of patients (%)	13 (52.0)
Documented resolution or significant improvement in symptoms, No. of Patients (%)	16 (64.0)

Abbreviation: PPI, proton pump inhibitors.

Twelve (48%) patients experienced additional ICI-related adverse events aside from gastrointestinal side effects being most common: colitis (*n* = 8, 32%), hepatitis (*n* = 2, 8%), and pancreatitis (*n* = 1, 4%). Other adverse events seen were arthritis (*n* = 2, 8%) and nephritis (*n* = 1, 4%).

Twenty-four (96%) received acid suppression as the initial treatment with proton pump inhibitors used in all cases with concern for melena on clinical presentation. Nine (36%) of patients also received steroids with an initial median dose of prednisone 75 (20-80) mg. No patients received biologic therapy for gastritis. On follow-up visits within 3 months of the initial presentation, 16 (64%) had documented complete resolution of symptoms. Thirteen (52%) were able to resume immunotherapy. Of note, 52.9% of patients on whom follow-up data is available (*n* = 17) had repeat endoscopic exams and 29.4% (5/17) had recurrent gastritis symptoms.

Immunotherapy was not resumed in 12 (48%) patients. We found only 1 (4%) case where the ICI-associated gastritis prohibited re-initiation of immunotherapy despite treatment with multiple courses of steroids and PPI and repeat endoscopy. Biologic therapy was not trialed in this patient for gastritis symptoms. Immunotherapy was not resumed in the other patients due to other various reasons. Three (12%) had other debilitating adverse events that precluded the restart of treatment including 2 cases of colitis and 1 case of suspected neurotoxicity from the medications. Two (8%) died before treatment could be restarted soon after gastritis diagnosis from metastatic disease and its complications and 1 (4%) transitioned to hospice. Three (12%) patients had disease progression despite immunotherapy and 2 (8%) patients opted to stop treatment.

### Endoscopy and Histology

On review of endoscopy reports, common findings were erythema 22 (88%), edema 13 (52%), and friability 12 (48%). Other features described were erosions 9 (36%), signs of bleeding 8 (32%), ulcerations 5 (20%), granularity 5 (20%), and atrophy 1 (4%). Inflammatory findings were reported to be focal in 9 (36%) patients.

**Figure 1.**
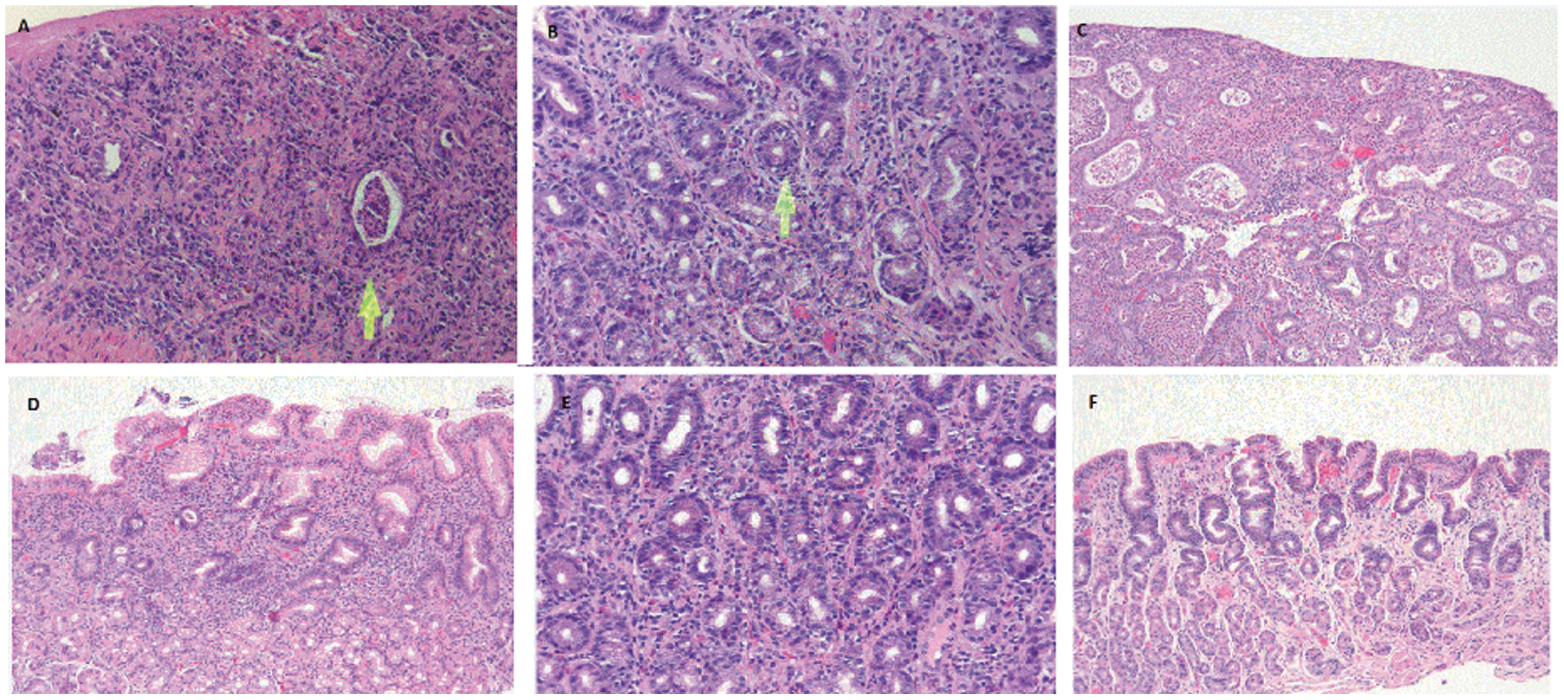
**A**. Chronic active gastritis: This case exemplifies a severe chronic active gastritis with extensive surface ulceration. Residual glands are present (arrow) and reveal neutrophilic microabscesses. The lamina propria is replaced by mixed inflammatory infiltrate including numerous plasma cells (H&E stain, 200×). **B**. Focally enhanced gastritis. The sections show lamina propria expansion by plasma cells, scattered lymphocytes, and eosinophils. There is neutrophil mediated injury (arrow). In addition, the surrounding glands demonstrate patchy intraepithelial lymphocytosis (H&E stain, 200×). **C**. Acute gastritis: gastric mucosa with florid neutrophilic mediated injury, including neutrophilic micro-abscesses and surface erosion (H&E stain, 10×). **D**. Chronic inactive gastritis: superficial band-like chronic inflammatory infiltrated in the lamina propria with foci of neutrophilic mediated injury, characteristic of chronic inactive gastritis (H&E stain 10×). **E**. Lymphocytic gastritis: prominent intraepithelial lymphocytes accompanied by a mild infiltrate in the lamina propria (H&E stain, 20×). **F**. Reactive gastropathy: marked reactive mucin loss in the foveolar epithelium with corkscrewing of gastric pits, capillary congestion and sparse lymphocytes and plasma cells in the lamina propria (H&E stain, 10×).

Histology slides for 14 patients were re-reviewed by a specialist gastrointestinal pathologist. Thirteen (92.9%) cases showed signs of mucosal injury. Individual details of the clinical characteristics of the 13 patients are presented in Table 4 and their histology is presented in Table 5. The overall pattern of injury was described as chronic gastritis with or without activity in 5 (5%), reactive gastropathy in 4 (28%), focally enhanced gastritis in 2 (14%), lymphocytic gastritis (7%), and acute gastritis in 1 (7%) patient (Fig. 1). Biopsy reports for three other patients where slides were not available for re-examination were suggestive of chronic active gastritis. Of the 14 available slides that were re-examined, the severity of inflammation was described as mild in 4 (28.6%) and severe in 2 (14.3%) patients. Excluding the lymphocytic gastritis case, intraepithelial lymphocytes (IELs) were present in 4 additional cases (28%) of patients. It was mostly present in a patchy distribution in the deeper glands and noted in the background of other patterns of injury described (reactive gastropathy, acute gastritis, focally enhanced gastritis, and chronic gastritis). Another prominent feature often seen was apoptosis, present in 7/14 (50%) cases. Lamina propria inflammation seen in 11/14 (78.6%) patients was mainly composed of plasma cells and was described as mild (*n* = 5, 35.7%), moderate (*n* = 5, 35.7%), and severe (*n* = 1, 7.1%). Lymphoid aggregates were present in only 2 (14.3%) patients. Rare eosinophils were identified in the lamina propria of 7 (50%) cases as well. Frank ulceration was noted in a single case on a biopsy where the endoscopy had revealed moderate inflammation and atrophic mucosa with erythema. No specimens had morphologic evidence of viral inclusions.

**Table 4.** Clinical and endoscopic characteristics of the 13 patients where biopsy slides were reviewed again for the purpose of this study.

Demographics age/race/sex	Cancer	Immunotherapy	Symptoms	Endoscopy	Treatment	Overall Outcome
60/W/M	Squamous cell lung	Nivolumab	Nausea/vomiting/abd pain	Erythematous mucosa/edema/ulcerations	PPI/prednisone 80	Died
74/W/F	Adenocarcinoma lung	Nivolumab	Nausea/pain/fatigue	Erythematous mucosa/edema/friability	PPI	Resolved
74/B/M	Urothelial	Pembrolizumab	Abd pain/melena	Erosions/ulcerations	PPI/carafate/prednisone	Resolved
76/W/F	Squamous cell lung	Nivolumab	Nausea/vomiting	Erythematous mucosa/edema/granularity	PPI/carafate	Immunotherapy cont.
59/O/M	Adenocarcinoma lung	Pembrolizumab	Nausea/vomiting/abd pain	Erythematous mucosa/edema/friability	PPI/carafate/prednisone 80	Unable to resume
75/W/M	Adenocarcinoma lung	Nivolumab	Nausea, melena, anorexia	Erythematous mucosa/edema	PPI	Immunotherapy cont.
65/W/F	Renal	Nivolumab	Abd pain/melena	Erythematous mucosa/erosions/ulcerations	PPI/carafate	Immunotherapy cont.
50/W/M	Squamous cell lung	Durvalumab	Abd pain/nausea	Erythematous mucosa/edema/friability	PPI/prednisone 40 mg	Disease progression
50/W/M	Melanoma	Nivolumab	Nausea, vomiting, abd pain, melena	Erythematous mucosa/erosions/edema	PPI/carafate	Resolved
79/W/M	Melanoma	Ipilimumab/nivolumab	Nausea/vomiting/abd pain	Erythematous mucosa/friability	PPI/prednisone 50 mg	Resolved
51/W/M	Non-Hodgkin’s lymphoma	Nivolumab	Bloating/abd pain	Erythematous mucosa/edema/erosions	PPI	Resolved
43/W/F	Melanoma	Pembrolizumab	Abd pain/nausea	Erythematous mucosa/edema/friability	PPI/prednisone 70 mg	Resolved
69/W/M	Mesothelioma	Ipilimumab/Nivolumab	Nausea/vomiting	Erosions/Ulcerations	PPI/prednisone 40 mg	Resolved

Abbreviations: W, white; O, other; B, black; M, male; F, female; PPI, proton pump inhibitor.

**Table 5.** Histological characteristics of the 13 patients whose slides were reviewed for the purpose of this study and showed inflammation.

Demographics	Predominant injury pattern (acute/chronic)	Intraepithelial lymphocytes	LP inflammation (none/mild/moderate/severe)	Apoptosis (present/absent)	Lymphoid aggregates (absent/present)	Eosinophils
60/W/M	Chronic active gastritis with ulcer	Absent	Severe (plasma cell)	Absent	Absent	Present (rare)
74/W/F	Chronic gastritis with lymphocytic gastritis pattern	Present (in deeper glands and surface)	Mild (plasma cells)	Present	Absent	Present (rare)
74/B/M	Reactive gastropathy	Present (rare in deep glands)	Absent	Present	Absent	Absent
76/W/F	Reactive gastropathy	Absent	None	Absent	Absent	Absent
59/M/O	Focally enhanced gastritis	Absent	Moderate (plasma cells)	Present	Absent	Absent
75/W/M	Reactive gastropathy	Present (in deeper glands)	Mild (plasma cells)	Present	Absent	Absent
65/W/F	Lymphocytic Gastritis	Present	Moderate (plasma cell)	Present	Present	Present (rare)
50/W/M	Chronic inactive gastritis	Absent	Mild (plasma cells)	Absent	Absent	Absent
50/W/M	Chronic gastritis	Absent	Moderate (plasma cells)	Absent	Absent	Present
79/W/M	Chronic gastritis with focal IM	Absent	Moderate (plasma cell)	Absent	Present	Absent
51/W/M	Focally enhanced gastritis	Absent	Mild (plasma cells)	Absent	Absent	Present (rare)
43/W/F	Acute gastritis	Present	Moderate (neutrophilic and plasma cells)	Present	Absent	Present (rare)
69/W/M	Reactive gastropathy	Absent	Mild (plasma cells and eosinophils)	Present	Absent	Present

## Discussion

We describe one of the largest case series to date of patients who develop symptoms attributed to upper gastrointestinal injury following the administration of ICI therapy. We found a variable number of infusions (1-30) of ICI therapy prior to symptom onset in our cohort. Nausea, vomiting, abdominal pain, and melena were the commonly experienced symptoms by patients. An endoscopy examination revealed erythema, edema, and friability. Following treatment with PPI and steroids, over half of the patients had documented complete resolution of symptoms within 2 months. Most patients in our cohort were able to resume ICI therapy following treatment for gastritis.

Histology review revealed chronic active gastritis and reactive gastropathy as the predominant patterns of mucosal injury seen in our cohort. IEL and apoptosis were prominent features in many of the cases. IELs were present mostly in the deeper glands and aside from one case of lymphocytic gastritis, were also seen with other patterns of inflammation: reactive gastropathy, acute gastritis, focally enhanced gastritis, and chronic gastritis. Lamina propria inflammation was mostly described as mild to moderate with one patient having severe inflammation in our cohort. Apoptosis was also described in half of the cases reviewed. Lymphoid aggregates were relatively rare and only seen in 2 patients.

Our study complements recent reporting of 3 case series over the last 2 years on this subject with some important distinctions as described below. Johncilla et al describe a series of 12 patients from 3 major tertiary care referral centers who underwent gastric biopsies for concern of ICI therapy-associated gastritis.^9^ Most common pattern of injury seen was chronic active gastritis in two-thirds of their patients compared to 7 patients in our study. They discovered a strong association of ICI gastritis with IELs and apoptosis in 7 out of 12 patients. IELs were present in 5 patients in our study and were seen with all patterns of injury. We also saw apoptosis in 7 patients in our study out of the 14 that were re-examined.

Zhang et al report a case series where 22 patients with upper gastrointestinal biopsies showed inflammation following ICI therapy.^10^ The most common pattern of inflammation was gastric peri-glandular inflammation in 15 patients which is similar to focally enhanced gastritis described in our study. Similar to us, chronic diffuse inflammation and reactive gastropathy patterns of injury were seen in 5 individuals each in their cohort. Interestingly, they report findings of granulomas in 12 of their patients more commonly in the group receiving combined anti-CTLA4 and PD1-PDL1 inhibitors. Commonly seen characteristics in our studies such as IELs and apoptosis were not seen in their study. These differences can be attributed to many factors including treatments used for gastritis prior to biopsy which was not described in their study.

Another case series published by Irshaid et al report 7 individuals showing gastritis features on histology following ICI therapy and compared them to biopsies of 8 patients diagnosed with *H. Pylori* gastritis.^13^ They found the pattern of inflammation to be similar with some key differences. They found prominent apoptosis in 5 and IELs in all 7 individuals with ICI-related gastritis consistent with our study and previously mentioned case series. We also found concomitant colitis in 32% of our patients. Diffuse gastrointestinal inflammation has also been demonstrated in a study by Tang et al, where 55% of patients who underwent both EGD and colonoscopy had endoscopic evidence of both upper and lower gastrointestinal inflammation.^11^

As described in our cohort and studies by Johncilla et al, Zhang et al, and Irshaid et al, a common histological pattern of mucosal injury is chronic active gastritis with lymphoplasmacytic infiltrate in individuals receiving ICI therapy. This pattern of injury has traditionally been described with *H. Pylori* infection. Rigorous assessment for this microorganism should be performed on all biopsied samples to rule out *H. Pylori* as the etiology in patients suspected of ICI gastritis. The 2 might look similar, however, usually *H. Pylori* gastritis is associated with neutrophilic or mixed infiltrate compared to predominantly lymphocytic infiltrate of ICI therapy-associated gastritis.^14^ Mixed infiltrate was seen in only 1 patient in our series while all others had lymphoplasmacytic infiltrates. *H. Pylori* gastritis is also associated with marked mucosal injury and ulcerations not often seen in our patient population.^15^

Cytomegalovirus (CMV)- and Epstein-Barr Virus (EBV)-associated gastritis are relatively rare but have also been described with lymphoplasmacytic infiltrate in the lamina propria with some glandular invasion. CMV gastritis is often associated with immunosuppression such as human immunodeficiency virus (HIV),^16^ while EBV has been described in immunocompetent individuals as well.^17^ They can be distinguished from ICI gastritis on histology by the presence of atypical lymphocytes with prominent nucleoli.^18^ Immunohistochemistry and PCR testing can be pursued to confirm the diagnosis if suspicion for viral-associated gastritis is high.^19^ Timing of symptom onset can often guide to the correct diagnosis where histological findings overlap between different etiologies of gastritis. Median time to symptom onset in our cohort was 2 weeks from the last infusion of immunotherapy but ranged from 3 days to 12 weeks indicating high variability. Similarly, a median number of immunotherapy transfusions was 4 prior to the onset of gastritis but also ranged from 1 to 30. Therefore, while histology can be extremely helpful in narrowing the differential, other etiologies of gastritis need to be eliminated prior to attributing presentation to ICI therapy due to the impact of this diagnosis on cancer treatment.

Focally enhanced gastritis pattern seen in a subset of our patients bears resemblance to gastritis secondary to inflammatory bowel disease, mainly Crohn’s disease although it has been described in ulcerative colitis as well.^20^ Another differential for this pattern can be gastric sarcoidosis.^21^ However, epithelioid granulomas characteristic of these 2 conditions were not seen in our population differentiating ICI gastritis from these 2 etiologies. The role of histiocytes governed by tumor necrosis factor (TNF) in this pattern of inflammation and similarity to Crohn’s disease can favor use of anti-TNF agents such as infliximab for treatment of these patients. The 2 patients in the cohort of Johncilla et al who received infliximab did report improvement in their symptoms. Current guidelines recommend use of selective immunosuppressive therapy with infliximab^22^ and vedolizumab^23^ for immune-related colitis following 4-6 weeks of steroid treatment. No such recommendation exists for immune-related gastritis. Successful treatment of upper gastrointestinal irAE with biologics has been reported. Badran et al report a case series of 5 patients who received contiguous infliximab and ICI therapy for various malignancies. Three of their patients had gastritis findings on endoscopy along with colitis.^24^ Repeat endoscopies showed resolution of inflammation and restaging did not show progression of malignancy.

This report hopes to contribute to identification and management of this pathology by providing data on a large collection of cases describing gastritis in the setting of ICI therapy. We provide a complete clinicopathological picture rather than focusing on one aspect alone for clinicians to recognize disease pattern of ICI gastritis. Like other researchers, we have detected that the disease spectrum in terms of presentation, clinical course, and histopathology varies widely. We also summarize previously published data with regard to histological characteristics to provide important diagnostic clues for ICI gastritis using a similar framework as other investigators.

There are several limitations to our study. Due to the retrospective design, we were unable to report long-term outcomes for all patients in our cohort. We only had access to 17 (14 histologic slides reviewed and 3 reports) out of 25 histology reports. Hence, we can only describe the clinical and endoscopic characteristics of the other 8 patients. We also acknowledge that gastritis seen in patients included in this study can be attributed to other etiologies, including NSAID use, chemotherapy, and radiation. We have described these variables in our results and only included patients who presented within 12 weeks of immunotherapy administration. We also excluded cases where biopsy showed luminal malignancy or *H. Pylori* gastritis.

To conclude, ICI therapy-associated gastritis often occurs with other gastrointestinal toxicities. Common patterns of inflammation are chronic active gastritis, reactive gastropathy, and focally enhanced gastritis. Steroids often lead to improvement in symptoms and allow the resumption of ICI therapy. Therefore, it is important to recognize this complication early based on clinical presentation with help from endoscopy and histology. Future studies should focus on mechanisms to prevent this complication of therapy and better means of treatment.

## Data Availability

The data underlying this article will be shared on reasonable request to the corresponding author.
